# Rare case report: a 26-year-old man with Eales’ disease

**DOI:** 10.22336/rjo.2023.34

**Published:** 2023

**Authors:** Sandra Bleidele, Igors Solomatins, Aīda Macijevska

**Affiliations:** * Dr. Solomatin Eye Center, Riga, Latvia

**Keywords:** Eales’ disease, periphlebitis, tuberculosis, biological drugs

## Abstract

**Purpose:** To report the case of a 26-year-old male with bilateral Eales’ disease that led to total blindness in the left eye and legal blindness in the right eye in a short time.

**Methods:** A total clinical systemic examination, computed tomography, magnetic resonance imaging, genetic testing, and optical coherence tomography were performed in the reported case.

**Results:** The eye condition was managed by scatter laser treatment, Anti-VEGF injections, anterior chamber paracentesis and trabeculectomy. Non-steroidal eye drops, as well as prostaglandin analogues, beta-blockers, and carbonic anhydrase inhibitors, have been used as local treatment. Systemic treatment included an intravenous methylprednisolone course, oral corticosteroids, azathioprine, mycophenolate mofetil and a total amount of 12 Anti-VEGF injections.

**Conclusion:** Despite the aggressive treatment with oral steroids, immunosuppressants, and anti-VEGF injections, there were many exacerbations, and remission was not achieved. As a result, aggressive neovascular glaucoma developed, which led to total blindness in the left eye and legal blindness in the right eye.

**Abbreviations: **HLA = human leukocyte antigens, Anti-VEGF = vascular endothelial growth factor inhibitors, BCVA = best corrected visual acuity, FA = fundus angiography, HBsAg = hepatitis B surface antigen, Anti-HCV = hepatitis C antibodies, TPHA = Treponema Pallidum hemagglutination assay, PCR = polymerase chain reaction, HSV = Herpes simplex virus, VZV = Varicella zoster virus, CMV = cytomegalovirus, IOP = intraocular pressure

## Introduction

Eales’ disease is an idiopathic peripheral retinal vasculature wall inflammation of unknown etiology, leading to peripheral retinal ischemia and neovascularization, which can be complicated with neovascularization and further vitreous body hemorrhage. It is mainly observed in young healthy males and is common in the Indian subcontinent. Eales’ disease is connected to Mycobacterium tuberculosis infection, caused by an immune response to the tuberculin protein, but human leukocyte antigens (HLA type) may also be involved. However, the etiology of Eales’ disease appears to be multifactorial and still unclear [**1**,**2**].

The pathology of disease may be divided into the following stages: inflammation, ischemia and proliferation stage, complicated with neovascularization and recurrent vitreous body hemorrhage. Treatment with oral steroids is provided in the early acute inflammatory phase, which is characterized by periphlebitis. Laser scatter is the treatment of choice to prevent neovascularization and establish later proliferative stages. In case of insufficiency of laser scattered therapy, intravitreal injections of vascular endothelial growth factor inhibitors (Anti-VEGF) are effective in preventing further neovascularization [**1**,**2**].

The most common complications of Eales’ disease are recurrent vitreous body hemorrhages, retinal detachment, macular edema, choroidal epiretinal membrane, cataract and neovascular glaucoma [**1**,**3**].

## Case presentation

A 26-year-old male was consulted in the clinic with complains of decreased, blurred vision and recurrent black floaters, which spontaneously disappeared within one week. The onset of symptoms was in 2019. Initially, floaters were only observed in the left eye, and then emerged in the right eye. 

**Fig. 1:**
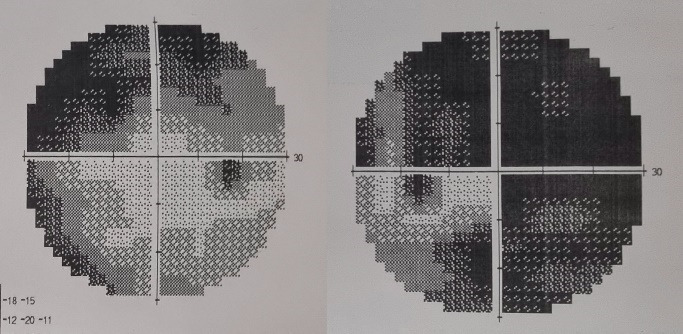
Visual fields demonstrate unconventional glaucomatous visual field defects and retinal defects caused by retinal pathology

At presentation (February 2021), his best corrected visual acuity (BCVA) was 0,16 in both eyes, with intraocular pressure of 19 mmHg in the right eye and 27 mmHg in the left eye. Anterior segment examination of the two eyes was completely normal. The visual field was carried out by Goldman perimetry (central 30-2 test) and showed unconventional glaucomatous visual field defects and retinal defects caused by retinal pathology (**Fig. 1**). 

**Fig. 2:**
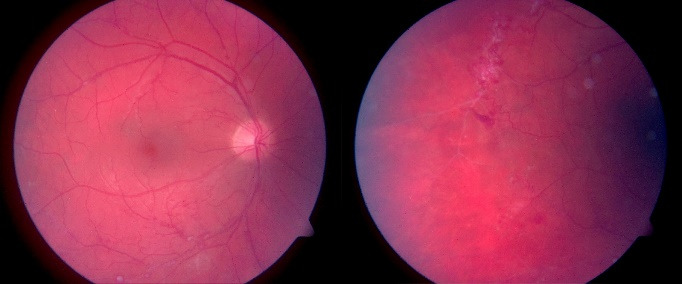
Color fundus photo of the right eye showing hemorrhages in the lower temporal zone; peripheral retinal neovascularization and area of non-perfusion

**Fig. 3:**
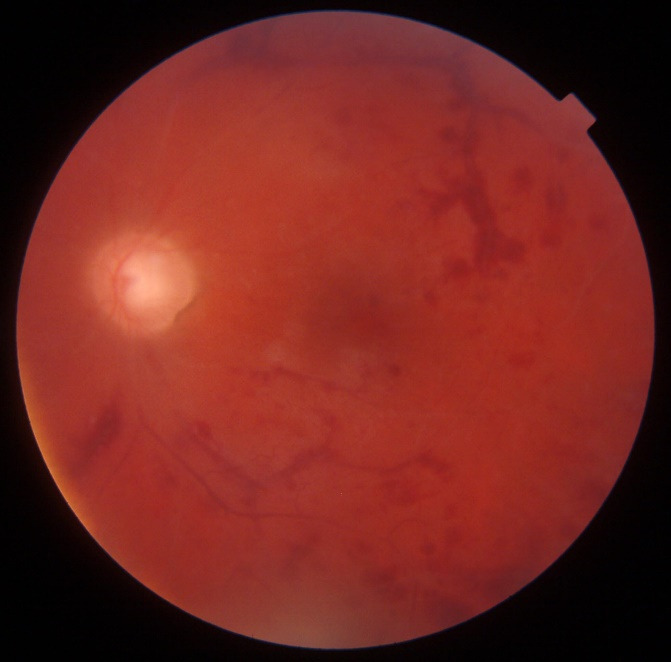
Color fundus photo of the left eye, showing pale, discolored optic nerve disc; narrowed and occluded small retinal arteries that are mainly displayed in the upper-temporal and the lower-temporal areas; the patient had significant retinal hemorrhages of various sizes and shapes

**Fig. 4:**
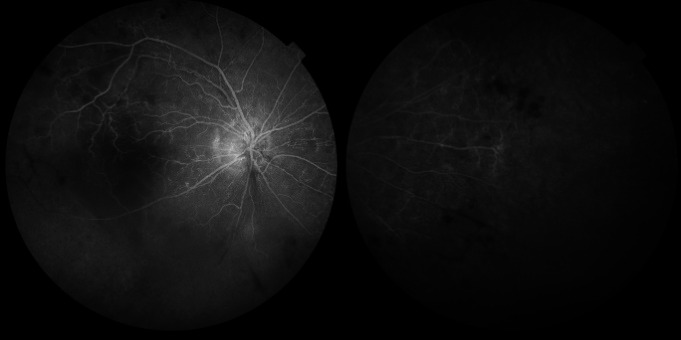
FA of the right eye

**Fig. 5:**
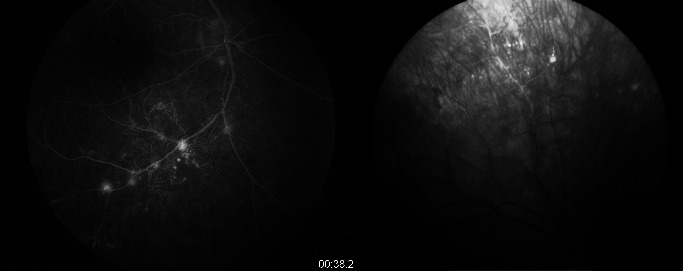
FA of the left eye, lower right and left

Dilated fundus eye examination of the right eye demonstrated peripheral retinal neovascularization and area of non-perfusion (**Fig. 2**), while the examination of the left eye demonstrated pale, discolored optic nerve disc, narrowed and occluded small retinal arteries, mainly in the upper-temporal and the lower- temporal areas, and multiple hemorrhages in different sizes and shapes (**Fig. 3**). FA of both eyes demonstrated sclerotic retinal vessels, a large area of ischemia, and increased retinal thickness or macular edema (**Fig. 4**,**5**).

After consulting an ophthalmologist, the patient was admitted to the hospital for a further diagnosis. Prior to this, the patient did not have any accompanying systemic signs or symptoms. Systemic examination was completely normal (complete blood count, erythrocytic sedimentation rate, blood sugar and coagulation). Infectious diseases (HBsAg, Anti-HCV, TPHA, Anti-HIV 1/ 2 and HIV 1 Ag, Toxocara canis IgG, Toksoplasma gondii IgM/ IgG and PCR for HSV, VZV, CMV) and autoimmune diseases, such as systemic lupus erythematosus (SLE), systemic sclerosis, etc., showed no pathological findings. Tuberculosis and sarcoidosis were excluded due to negative results of QuantiFERON-TB Gold test, serum angiotensin-converting enzyme (ACE), serum lysozyme and chest computed tomography. Magnetic resonance imaging (MRI) of the brain and orbit, with gadolinium, was done to evaluate the demyelinating lesions. By that time, he had been genetically tested for Familial exudative vitreoretinopathy (FEVR), which was also negative.

According to the patient’s medical records and further systemic examination, all infectious diseases and systemic disorders were excluded as the primary diagnosis. The diagnosis of Eales’ was based on the patient’s fundus examinations and paraclinical investigations. 

The eye condition was managed by systemic treatment, laser photocoagulation, Anti-VEGF injections, and later with trabeculectomy. The patient received treatment with intravenous methylprednisolone course followed by oral corticosteroids (32 mg per day). The quantity of oral corticosteroids was reduced to a minimally effective dose. 

**Fig. 6:**
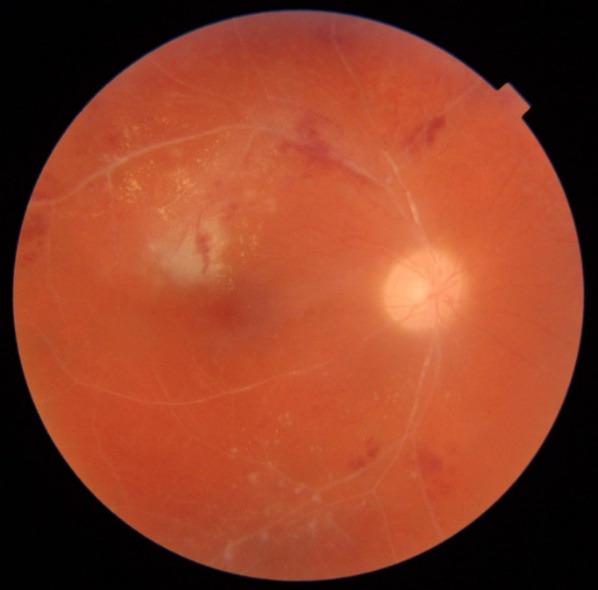
Color fundus photo of the right eye after one year of treatment, showing pale optic nerve disc; peripheral neovascularization with a ring of lipid exudation; sheathing of retinal vessels

Nevertheless, there were numerous exacerbations, and consequently immunosuppressive therapy with Azathioprine (AZA) was added (150 mg per day). During azathioprine treatment, the disease started to progress in the right eye (**Fig. 6**) and a vitreous hemorrhage occurred. Aggressive neovascular glaucoma with neovascularization of the iris (NVI) developed in the left eye. For that reason, AZA was substituted with Mycophenolate Mofetil (CellCept) in a dosage of 2 g per day.

**Fig. 7:**
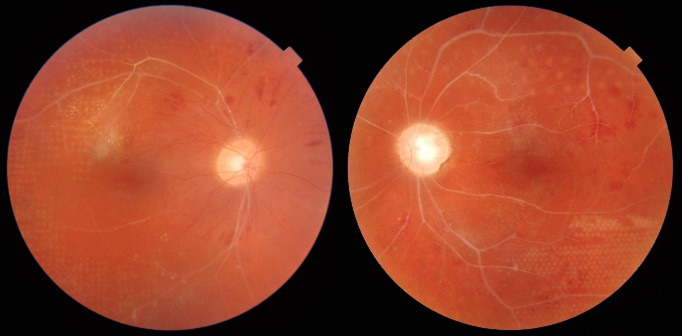
Fundus photographs showing scatter laser photocoagulation scars; A - right eye; B - left eye

The eye condition was managed by scatter laser therapy and Anti-VEGF injections (Avastin). A scatter laser treatment was carried out in both eyes to control ischemic areas of the peripheral retina and to reduce formation of new vessels (**Fig. 7**). In total, 12 Anti-VEGF injections were performed in both eyes.

The diseases rapidly spread to the right eye, aggressive neovascular glaucoma developing within a year and a half. In this case, we faced a resistance to medical treatment, being difficult to reach a target level of IOP, due to which, paracentesis of the anterior chamber was performed. However, IOP pressure was not appropriate, and he complained of ocular pain. To reduce IOP more effectively, sinus trabeculectomy was performed in both eyes. After surgery, the reduction of IOP was not enough in the right eye, and because of the sinus trabeculectomy, a shunt implant was performed in the right eye. 

Currently, he has decompensated open angle glaucoma, optic atrophy, retinal vascular sheathing, and mydriasis. His left eye is totally blinded, while the right eye is legally blinded (only hand motion). Now, he is administered medications such as eye drops - Cosopt, Brimonidine and Latanoprost. Oral steroids and immunosuppressive therapy were revoked because no improvement was observed during the therapy and no remission was achieved, and to reduce the risk of systemic side effects.

## Discussion

Eales’ disease is named after Henry Eales, who was the first to describe patients with recurrent vitreal and retinal hemorrhages in 1880, which were associated with constipation and epistaxis [**4**].

Although the etiology of Eales’ disease remains uncertain, it seems to be multifactorial and heterogeneous. Furthermore, its association with Mycobacterium tuberculosis, Mycobacterium fortuitum, and Mycobacterium chelonae exposure has been reported, causing a retinal autoimmune response [**4**,**5**]. Studies have shown that the tuberculin skin test (TST) was positive in 64% of patients suffering from Eales’ disease [**6**]. In addition, individuals predisposed to HLA (HLA B5, DR1, DR4) are more likely to have an autoimmune response, due to tissue damage caused by mycobacterial antigen [**5**].

Inflammatory occlusion of the retinal vasculature results in retinal hypoxia, triggering further inflammation. Various studies have shown that vascular endothelial growth factor (VEGF), chemokine monocyte chemoattractant protein (MCP-1), and interleukins (IL-6 and IL-8) are elevated in the vitreous body of Eales’ disease patients, especially in the proliferative stage [**4**,**7**]. 

In most cases, Eales’ disease is a diagnosis of exclusion. Many disorders can cause inflammation or occlusion of the retinal vascular system, and due to this, a careful systemic examination is necessary to rule out other diagnoses, which can mimic Eales’ disease. For example, RBVO (retinal branch vein occlusion), proliferative diabetic retinopathy, familial exudative vitreoretinopathy (FEVR), sickle-cell retinopathy and leukemia should be excluded as the primary diagnosis [**1**,**7**]. Eales’ disease mostly affects veins rather than arteries. For this reason, we need to exclude disorders affecting veins, like tuberculosis, sarcoidosis and syphilis [**1**].

The stage of Eales’ disease determines the further treatment options. There are various treatments such as observation, therapy with medical drugs, laser photocoagulation and surgery [**1**].

In case of active perivasculitis and a positive tuberculin test, it is recommended to start antitubercular therapy. Oral corticosteroids and empirical anti-tubercular therapy (ATT) can be considered in combination [**1**]. However, the use of ATT in the treatment of Eales’ disease is limited and unproven [**4**], causing multiple systemic side effects, and risk/ benefit analysis should be performed [**1**].

If presentation of Eales’ disease is unilateral, it is recommended to start treatment with periocular or intravitreal depot corticosteroids (triamcinolone acetonide or dexamethasone) to reduce the side effects of long-term systemic corticosteroids [**6**]. Systemics steroids (usually 1 mg/ kg body weight per day) are used in bilateral or severe cases to minimize the site of inflammation in Eales’ disease [**1**].

Once the proliferative stage is reached, peripheral laser scatter photocoagulation is the best option to border areas of non-perfusion and ischemic zones. The laser is contraindicated in acute stages of vasculitis, due to the release of more angiogenic factors, which aggravate neovascularization [**7**]. Like other vascular disorders, Eales’ disease has a good response to corticosteroids treatment. Vitrectomy is the choice of treatment in cases with recurrent vitreous body hemorrhages with or without retinal detachment [**1**].

This case was unique due to its rapid progression over a short period of time. The main complication in this case was the aggressive neovascular glaucoma, which led to optic atrophy and resulted with total blindness in the left eye and legal blindness in the right eye (only hand motion). Therefore, there was no improvement during medical therapy and remission was not achieved.

Unfortunately, in case of Eales’ disease, no documentation of biological drug treatment exists. Biological drugs are currently used for the treatment of vasculitis, such as anti-TNF-alpha agents (infliximab, etanercept, adalimumab, golimumab, and certolizumab), anti-interleukin (IL)-6-receptor antibody (tocilizumab), and anti-CD20 antibody (rituximab) [**6**,**8**,**9**]. Even so, we need more research on the use of biological drugs for Eales’ disease, which is aggressive and resistant to medical treatment. 

## Conclusion

Eales’ disease is a rare autoimmune disease, characterized by retinal periphlebitis, ischemia, and neovascularization. The etiology of disease is still poorly understood. A complete systemic and clinical examination should be performed to rule out other disorders mimicking Eales’ disease. 

This case report was unique and remarkable for its rate of progression, medical resistance, and complications. In this case, the steroids used, autoimmune therapy, anti-VEGF injection, scatter laser treatment and trabeculectomy were insufficient to stop the progression of the disease and remission was not achieved. The main complication at that moment was aggressive neovascular glaucoma, which led to optic atrophy and consequently to total and legal blindness. 

Unfortunately, the use of biological drugs, such as anti-TNF-alpha agents, anti-interleukin, and anti-CD20 antibody are not reported in the treatment of Eales’ disease. Further research should be carried out to improve the treatment outcomes of Eales’ disease.

**Conflict of Interest statement**

The authors state no conflict of interest.

**Informed Consent and Human and Animal Rights statement**

Informed consent has been obtained from the individual included in this study.

**Authorization for the use of human subjects**

Ethical approval: The research related to human use complies with all the relevant national regulations, institutional policies, is in accordance with the tenets of the Helsinki Declaration, and has been approved by the review board of Dr. Solomatin Eye Center, Riga, Latvia.

**Acknowledgements**

None.

**Sources of Funding**

None.

**Disclosures**

None.
